# The impact of health education interventions on oral health promotion among older people: a systematic review

**DOI:** 10.1186/s12877-023-04259-5

**Published:** 2023-09-11

**Authors:** Saeid Bashirian, Sahar Khoshravesh, Erfan Ayubi, Akram Karimi-Shahanjarini, Samane Shirahmadi, Parshang Faghih Solaymani

**Affiliations:** 1 Autism Spectrum Disorders Research Center, Hamadan University of Medical Sciences, Hamadan, Iran; 2 Department of Community Health Nursing, School of Nursing and Midwifery, Hamadan University of Medical Sciences, Hamadan, Iran; 3 Chronic Diseases (Home Care) Research Center, Hamadan University of Medical Sciences, Hamadan, Iran; 4 Cancer Research Center, Hamadan University of Medical Sciences, Hamadan, Iran; 5 Social Determinants of Health Research Center, Hamadan University of Medical Sciences, Hamadan, Iran; 6 Department of Community Oral Health, School of Dentistry, Dental Research Center, Hamadan University of Medical Sciences, Hamadan, Iran

**Keywords:** Older people, Caregiver, Oral health, Intervention, Health education

## Abstract

**Background:**

One of the most common pathologic changes in older people is oral and dental problems. The oral health of older people is a public health concern. Promotion of good oral health for this cohort will have beneficial impacts on the longer-term quality of life. This study aimed to identify the types of health education interventions for the oral health of older people and to determine their effects on the oral and dental health of older people.

**Methods:**

Potential articles were retrieved from four electronic databases (PubMed/Medline, Scopus, Web of Sciences, and Embase) up to 31 September 2022 in English without limit of time. Experimental and quasi-experimental interventional studies investigating the impact of educational interventions on oral and dental health among older people over 60 years old in both sexes were considered. The quality assessment tool was the Effective Public Health Practice Project (EPHPP).

**Results:**

In the initial search, 1104 articles were retrieved. Finally, according to the inclusion criteria, 23 studies (seventeen randomized controlled trials (RCT) and six quasi-experimental studies) were reviewed. In this review, educational interventions for older people and their caregivers are classified. Theoretical frameworks were used in only three interventions related to older people. Outcome measures were both self-reported and objective measures. Fifteen of the included studies were of moderate quality.

**Conclusion:**

This review provides evidence that the use of oral and dental health educational interventions was effective in improving the oral health of older people. Educational interventions were carried out both among older people and among their caregivers. Although a variety of interventions were used in the reviewed studies, more lectures were used in the interventions related to older people. In the interventions related to caregivers, in addition to lectures, practical education was also used. It is recommended to perform higher quality studies for assessing the effectiveness of interventions in this field.

**Supplementary Information:**

The online version contains supplementary material available at 10.1186/s12877-023-04259-5.

## Introduction

The improvement of living conditions and the increase in life expectancy have led to the phenomenon of aging in societies [1], in such a way that it has become one of the challenges of public health all over the world [2]. According to the report of the World Health Organization, between 2015 and 2050, the proportion of the population over 60 will almost double from 12 to 22% [3]. The aging process includes a natural course in which many physiological and psychological changes occur in the body [4]. Oral and dental problems are generally pathological processes that may also result from the aging process. These problems include tooth loss, dry mouth, gum disease, tooth decay, oral mucosa disorders, and chewing disorders [5]. These changes can affect the quality of life of older people [6].

One of the important concerns for public health is the improvement of older people's health, which can lead to an improved quality of life among them [7]. The evidence indicates that oral and dental health problems among older people have been given less attention compared to cardiovascular or neoplastic diseases [8, 9]. Oral and dental health means the health of the oral cavity and its related tissues. Good oral health facilitates a person for eating, speaking and social interaction [10]. Oral health-related quality of life (OHRQOL) is a complex concept that consists of four dimensions: functional factors, psychological factors, social factors, and experience of pain or discomfort [11]. Patients with poor oral and dental health may have lower mood, more life stress and reduced quality of life [12]. Some older people have many oral and dental problems that can negatively affect their physical or psychosocial health. For example, it can lead to a reduction in fruit and vegetable consumption in older people [13]. This nutritional style can cause nutritional disorders in older people [14, 15]. Often, older people with dentures complain of a wide range of problems including eating, social interaction, and communication, and these problems have a detrimental effect on their quality of life [16].

In recent years, in order to improve the oral health-related quality of life, attention has been focused on evaluating the effectiveness of oral health education programs. A number of systematic reviews have been conducted on the available evidence, the results of which have shown that oral health education can be effective in the short term in increasing knowledge and to some extent behaviors such as brushing teeth and healthy eating [17]. Considering the phenomenon of aging and the importance of the health and quality of life of older people, which is affected by various factors such as oral and dental hygiene, the importance of prevention and the need for appropriate interventions to improve the health of older people are felt. Therefore, this study aimed to identify the types of health education interventions and to determine their effects on oral and dental health in older people.

## Methods

This study was performed based on the Preferred Reporting Items for Systematic Reviews and Meta Analyses (PRISMA) guidelines [18]. This systematic review as approved by the Research Ethics Committee of Hamadan University of Medical Sciences (No. IR.UMSHA.REC.1400. 829).

### Search strategy

Potential articles were retrieved from four electronic databases (PubMed/Medline, Scopus, web of sciences, and Embase) up to 31 September 2022 in English without limit of time. The search strategy was developed using Medical Subject Headings (MeSH). We used the keywords of Wang et al.'s study as a basis [19]. The keywords were considered based on Population, Intervention, Comparison, Outcomes and Study design (PICOS) as a framework to formulate eligibility criteria in this study [20]. The search strategy for PubMed/Medline is described in Appendix 1.

### Population

Older people over 60 years old in both sexes without cognitive impairment/dementia were considered.

### Intervention

All interventional studies investigating the effect of educational interventions on oral and dental health were included in the study. These educational interventions could involve older people or their caregivers. The use of the theoretical framework in the reviewed studies was also investigated.

### Compare

Interventional studies with all types of comparatives were included in this study.

### Outcome

Promoting oral and dental health in older people was the first outcome. The second outcome was the quality of life related to oral health.

### Selection of studies

The results of initial searches were independently screened by two authors according to titles, abstracts, and full texts. Any disagreement among the researchers regarding the exclusion or inclusion of articles in the study was resolved with discussion. All searched articles in the initial search were entered into EndNote X8 software.

### Study eligibility

Experimental and quasi-experimental interventional studies investigating the impact of educational interventions on oral and dental health among older people over 60 years old in both sexes were considered. Descriptive, qualitative, review studies, letters and correspondences, editorials, conference proceedings and studies that consider oral and dental health along with other interventions to perform other health behaviors were excluded.

### Data extraction

Data was independently extracted by two authors (PF and SK). Any discrepancy was resolved through discussion. The extracted information included the following: first author (year), country, study design, study population (age, gender), study groups, description of intervention and control, and oral health main findings. In this study, the results of data extraction are independently presented based on the subjects of intervention (older people and caregivers). After completing the search in the mentioned databases, it was found that the educational interventions related to the oral and dental health of older people were carried out in two ways: directly (the target group was the older people themselves) and indirectly (the target group was the caregivers of older people). For this reason, the classification of studies was carried out by the research team in the current form in order to provide the possibility of comparison.

### Quality assessment tool

The included studies were independently evaluated by two authors using the Effective Public Health Practice Project (EPHPP) quality assessment tool [21].

This tool has six subscales including selection bias, study design, confounding, blinding, data collection methods, and withdrawals/drop-outs. Any disagreement among the researchers regarding the scoring of the quality assessment tool was resolved by discussion or by a third author. Inter-rater reliability was approved by Cohen’s Kappa coefficient. Cohen suggested the Kappa result be interpreted as follows: values ≤ 0 as indicating no agreement and 0.01–0.20 as none to slight, 0.21–0.40 as fair, 0.41– 0.60 as moderate, 0.61–0.80 as substantial, and 0.81–1.00 as almost perfect agreement [22]. None of the studies were excluded based on quality assessment results.

## Results

### Results of the searched studies

**Fig. 1:**
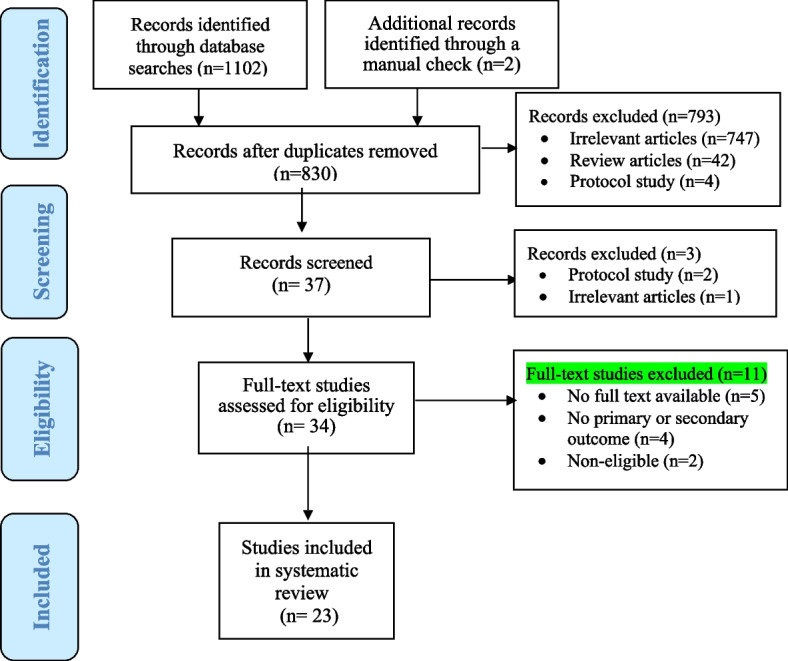
PRISMA flow diagram of the systematic review and meta-analysis selection process

1102 articles were retrieved from the four electronic databases; PubMed/Medline (*n* = 122), Scopus (*n* = 580), Web of Sciences (*n* = 297), and Embase (*n* = 103). To minimize retrieval bias, the inclusion criteria were manually checked for additional eligible documents that could have been missed during the mentioned database and grey literature search (*n* = 2). Finally, 1104 articles were retrieved in the initial search. Duplicated items were identified using EndNote X8 software and manually removed from the articles file. After removing duplicates, 830 articles remained. Of these articles, 793 articles were excluded because they were not in line with the objectives of the study. Then, 37 articles were screened. Three articles were excluded from the screening stage. In the next step, the full texts of 34 eligible articles were assessed. Finally, 23 articles were reviewed in this systematic review (Fig. 1). Details of the included final studies are presented in Tables 1 and 2 based on the subjects of intervention (older people and caregivers).

**Table 1:** Effectiveness of intervention to oral health promotion among older people

**Row**	**First author (year)**	**Country**	**Study** **design**	**Study population** **(Age, gender)**	**Study groups**	**Description of intervention and control/**	**Oral health main findings**	**Theoretical framework**
1	Keyong E (2019) [23]	Thailand	RCT	All: *n* = 162 Age: 60–74 y Female: *n* = 79 Male: *n* = 77	I: *n* = 79 C: *n* = 77	**Intervention**: Older people in intervention group received oral hygiene care educational programs based on the HBM theory **Baseline**: Educations was about health risk, disease severity, and benefit of behavior changes, and then oral health‑related knowledge. Education was conducted by two trained nurses for 4–5 groups in 30–35 min sessions **Month 1**: Follow‑up oral health behavior and a review of oral health‑care skills and knowledge **Month 3**: Phone calls to ensure compliance and to review oral health‑care skills and knowledge **Control**: Only regular oral health promotion	After 6 months, perceived severity, perceived severity, perceived benefits, perceived barriers and Self‑efficacy were statistically significant between two groups (*p* < 0.05). Moreover, Plaque, index score, gingival index score and clinical attachment loss (*p* < 0.05) **Conclusion**: This oral health promotion program improved oral health perception, behavior, and oral health status of the older people	Health Belief Model (HBM)
2	Ki JY (2021)	South Korea	RCT	All: *n* = 46 Age: 65–74 y Female: *n* = 20 Male: *n* = 20	I: *n* = 24 C: *n* = 22	**Intervention**: Older people in intervention group received oral health education using a mobile app (OHEMA) **Baseline**: Educations was about customized oral health care management, oral exercises, and intraoral and extraoral massage methods for 50 min/session, once a week, for 6 weeks **6 weeks**: follow up oral health behavior and a review of oral health care skills and knowledge **Control**: Did not undergo any oral hygiene education or OHEMA	After 6 weeks, tongue pressure increased, subjective oral dryness, un stimulated salivary flow rate were statistically significant between two groups (*p* < 0.001) **Conclusion**: OHEMA appears to be a useful tool for oral health education for the older people as it improved the SWAL-QoL, with increased tongue pressure and reduced oral dryness	
3	Lee KH (2020) [33]	South Korea	Quasi-experimental	All: *n* = 120 Age: > 65 y Female: *n* = 87 Male: *n* = 15	I_1_: *n* = 36 I_2_: *n* = 35 C: *n* = 31	**Intervention (Intervention group II):** Older people in intervention group received oral health education program using a workbook, immediately after the lecture **Baseline:** Oral health education was about oral health knowledge, oral health recognition, O’Leary index, tongue coating index; at once a week for 5 weeks **6 weeks**: Follow-up oral health behavior and a review of oral health care skills and knowledge **Control (Intervention group I):** Only received lectures on oral health education	After one-week, significant increase oral health knowledge, oral health recognition, decrease on the O’Leary index and tongue coating index; in both intervention groups **Conclusions**: The oral health education program using the workbook increased knowledge and recognition of oral health and lowered the O’Leary and tongue coating indexes. Hence, the use of a workbook may facilitate the effectiveness of oral health education for older people	
4	Lee KH (2021) [25]	South Korea	RCT	All: *n* = 73 Age: ≥ 65 y Female: *n* = 62 Male: *n* = 11	Non-app use: *n* = 25 App use: *n* = 22 C: *n* = 26	**Intervention**: **(App use group):** received oral health education using a smartphone app and workbook activities **Baseline**: Oral health education was about oral health knowledge, oral health recognition, O’Leary index, tongue coating index; twice a week for five weeks **6 weeks**: Follow up oral health behavior and a review of oral health care skills and knowledge **Control** (**Non-app use group**): Control group received lecture-based oral health education using power point presentations and participated in workbook activities	After 6 weeks, in non-app use group: significant increase oral health knowledge, decrease on the O’Leary index, tongue coating index In app use group: significant increase oral health knowledge, oral health perception, decrease on the O’Leary index, tongue coating index. Interaction between time and group was significant only in tongue coating variable **Conclusion**: The smartphone app developed in this study carries the possibility to convey informative content for oral health education among older adult	
5	Marino RJ (2016) [34]	Australia	Quasi experimental	All: *n* = 75 Age: ≥ 55 y Only female	I: *n* = 75	**Intervention:** Intervention group received oral health education based on the ORHIS (Oral Health Information Seminars/Sheets) Model and involved computer interaction with six oral health presentations (web based) **Baseline**: Oral health education was oral health attitudes, knowledge, self-efficacy, self-reported oral hygiene practices at twice a week for five weeks **After the intervention**: Follow up oral health behavior and a review of oral health care skills and knowledge **Control**: There was no control group	After the intervention, significant improvements in oral health attitudes, knowledge, self-efficacy, self-reported oral hygiene practices (*p* < 0.05) **Conclusion**: The e-ORHIS approach was successful in improving oral health knowledge, attitudes and self-efficacy. As such, it represents a helpful approach for the design of (oral) health interventions in older adults	Social Cognitive Theory (SCT)
6	Ohara Y (2015) [26]	Japan	RCT	All: *n* = 47 Age: > 65 y Gender is not mentioned	I: *n* = 21 C: *n* = 17	**Intervention**: Older people in intervention group received oral hygiene instruction, oral functional exercise and salivary gland massages, via lectures **Baseline**: Educations were about oral hygiene instruction, facial and tongue muscle exercise, and salivary gland massage. Education was conducted every 2 weeks for 3 months' six 90-min sessions **After the intervention:** Follow up oral diadochokinetic of articulation, swallowing, taste threshold and salivary flow rate **Control**: Oral health check-up	After the intervention, resting salivation, the second and third cumulated Repetitive Saliva Swallowing Test times, significantly improved The threshold for bitterness significantly lowered in the intervention group, compared with control group after 3 months (*P* < 0.05) **Conclusion**: The educational program targeting oral function improvement is effective among the independent older population	
7	Powell LV (1999) [27]	USA	RCT	All: *n* = 297 Age: > 60 y Female: *n* = 163 Male: *n* = 134	G_1_: *n* = 55 G_2_: *n* = 48 G_3_: *n* = 52 G_4_: *n* = 52 G_5_: *n* = 55	**Intervention**: Older people in five intervention groups received different caries-preventive strategies on caries progression **Baseline** **Group1**: Group1 received usual care from a public health department or a private practitioner **Group2**: Group2 received an educational program of 2 h duration implemented twice a year **Group3**: Group3 received the educational program plus a 0.12% chlorhexidine rinse weekly **Group4**: Group4 received the education and chlorhexidine interventions and a fluoride varnish application twice a year **Group5**: Group5 received all the above interventions as well as scaling and root planning every 6 months throughout the 3-year study **3-year**: Main outcome measures were oral health behavior and a review of oral health‑care skills and knowledge **Control**: There was no control group	**After 3-year,** groups that received usual intraoral procedures (groups 3, 4, and 5) had a 27% reduction for coronal caries events (p = 0.09) and 23% for root caries events (p = 0.15), when compared to the groups that received no intraoral procedures (groups 1 and 2) **Conclusion**: The caries progression in this lower-income, ethnically diverse, older population is relatively high. Simple preventive measures (education, chlorhexidine rinses, fluoride varnishes, root planning) result, at best, in only a moderate reduction in caries development	
8	Saengtipbovorn S (2014) [35]	Thailand	Quasi-experimental	All: *n* = 132 Age: > 60 Female: *n* = 85 Male: *n* = 47	I: *n* = 66 C: *n* = 66	**Intervention**: Older people in intervention group received lifestyle and oral health education program motivational interviewing (MI) **Baseline**: Educations was about type 2 diabetes complications, the prevention of general and oral health complications, the relationship between type 2 diabetes and oral complications, and oral health care, in 20 min; by trained nurse practitioners **Month 3**: Main outcome measures were: glycosylated hemoglobin (HbA1c), fasting plasma glucose (FPG), body mass index (BMI), periodontal status, knowledge, attitude and practice of oral health and diabetes mellitus **Control**: Control group received a routine program in the diabetes clinic	After the 3 months, Participants in the intervention group had significantly lower glycosylated hemoglobin (HbA1c), fasting plasma glucose (FPG), plaque index score, gingival index score, pocket depth, clinical attachment level (CAL), and percentage of bleeding on probing (BOP) when compared to the control group **Conclusions:** The combination of lifestyle change and dental care in one program improved both glycemic and periodontal status in the older people with type 2 diabetes	
9	Schou L (1989) [29]	UK	RCT	All: *n* = 201 Age: 48–99 y Gender is not mentioned	*n* = 201	**Intervention**: Older people in intervention group received dental health education program **Baseline**: Educations were about dental health education program Education was conducted of the three programs 1) Active involvement of residents only, 2) Active involvement of staff only, 3) Active involvement of both staff and residents **Month 2**: Follow up oral health and oral hygiene **Control**: No educational program	**After 2 Month,** poor oral health and oral hygiene, high objective need for oral care but low perceived need **Conclusion:** The implications of the study arc that groups of older people need to be differentiated further so that only well and not confused older people participate in programs such as this and less well and confused the older people receive regular professional support with oral hygiene	
10	Shokouhi E (2020) [30]	Iran	RCT	All: *n* = 86 Age: > 60 Female: *n* = 38 Male: *n* = 48	I: *n* = 43 C: *n* = 43	I**ntervention**: older people in intervention group received variables of oral health related quality of the older people, based on adult learning theory **Baseline**: A training program comprised of a combination of in-person training (individual training and group discussion) and non-attendance training (sending educational messages) was prepared and submitted 15 min of individual training based on motivational interviewing methods researcher-made booklet and a dental modulate was used to improve individual education **Month 1**: Main outcome measures were oral health related life quality, and effectiveness of adult learning the **Control**: No educational program	After 1-month, educational intervention was significant in terms of overall oral health related quality of life and the overall effectiveness score of adult learning theory (*P* < 0.001). There was a significant difference between the two groups in terms of the mean change score of three physical, psychosocial, and pain dimensions following the educational intervention (*P* < 0.001) **Conclusion:** Education based on adult learning theory is recommended for improving oral health related quality of life among the older people	Adult Learning Theory
11	Tellez M (2019) [31]	USA	RCT	All: *n* = 180 Age: ≥ 55 y Female: *n* = 112 Male: *n* = 68	G_1_: *n* = 60 G_2_: *n* = 60 C: *n* = 60	**Intervention**: Older people in intervention group received oral health education based on the 3 programs: motivational interviewing, traditional oral health education, and standard of care **Baseline**: Patients were randomly allocated to TOHE, MI and SC groups The MI intervention was administered by a Public Health Dental Hygienist (PHDH) **1-year**: Main outcome measures were: oral health-related quality of life (OHRQoL), oral health self-efficacy (SE) and oral health knowledge (OHK, between three groups) **Control:** Control group received traditional oral health education, standard of care	After 1 year, in intervention group significantly improved oral health-related quality of life (OHRQoL), oral health self-efficacy (SE), oral health knowledge, compared to the control group (*P* = < 0.001) **Conclusion**: Findings from the study support the fidelity of this intervention and the improvement of all non-clinical outcomes after 12 months amongst the MI group	
12	Sun KT (2021) [36]	Taiwan	Quasi experimental	All: *n* = 129 Age: ≥ 60 y Female: *n* = 93 Male: *n* = 36	I: *n* = 72 C: *n* = 57	**Intervention**: Older people in intervention group received oral health education materials based on the easy (EZ) to read” concept **Baseline**: Health education course of approximately 30 min accompanied by PowerPoint slides **After intervention**: Main outcome measures were oral health literacy adult questionnaire complete **Control**: Control group received general text material	EZ to read material significantly improved total scores of oral health literacy (*p* < 0.001) **Conclusions**: Introducing the EZ to read model to the design of older adult health education material in rural areas significantly improved their oral health literacy	
13	Saengtipbovorn S (2015) [28]	Thailand	RCT	All: *n* = 132 Age: > 60 y Female: *n* = 85 Male: *n* = 47	I: *n* = 66 C: *n* = 66	**Intervention**: Older people in intervention group received oral health education **Baseline**: Intervention group attended 20-min lifestyle and oral health education, individual lifestyle counseling, application of a self-regulation manual, and individual oral hygiene instruction **At month 3:** The intervention group received individual lifestyle counseling and oral hygiene instruction. The intervention group received booster education every visit by viewing a 15-min educational video **After intervention **[3, 6 months]**:** Follow-up for glycemic and periodontal status **Control**: Control group received a routine program	**After the 6-month**, participants in the intervention group had significantly lower glycated hemoglobin, fasting plasma glucose, plaque index, gingival index, probing depth, and attachment loss when compared with the control group **Conclusion**: The combination of lifestyle changes and dental care in one program improved both glycemic and periodontal status in older patients with diabetes	
14	Zhang W (2013) [32]	China, Hong Kong	RCT	All: *n* = 266 Age: 60–89 y Female: *n* = 198 Male: *n* = 68	I_1_: *n* = 98 I_2_: *n* = 84 C: *n* = 84	**Intervention**: Older people in intervention groups [2, 3] received oral health education and silver diamine fluoride, oral hygiene instructions **Baseline**: group 2 received OHI and silver diamine fluoride (SDF) application annually, and group 3 was given OHI and SDF application annually, plus an oral health education (OHE) program every 6 months **After intervention**: Main outcome measures were new root caries surfaces, arrested root caries surfaces, active root caries surfaces **Control** (group 1): Control group received oral hygiene instructions (OHI) annually	**After 24 months**: Group3 had fewer root surfaces with new caries; and Group3 and group 2 had a great number of active root caries surfaces which became arrested compared with the control group (Group 1) *p* < 0.05)	

*Note: n* Number, *G* Group, *I* Intervention, *C* Control or Comparison, *RCT* Randomized Controlled Trial

**Table 2:** Effectiveness of interventions to oral health promotion of older people among their caregivers

Row	First author (Year)	Country	Study design	Study population (Age, gender)	Study groups	Description of intervention and control	Oral health main findings
1	Frenkel HF (2002) [37]	Bristol, UK	RCT	All: *n* = 322 Age: ≥ 16 y Female: *n* = 283 Male: *n* = 12	I: *n* = 166 C: *n* = 156	**Intervention**: Caregivers in intervention group received oral health care education program (OHCE) **Baseline**: A Health Promoter presented the intervention. Each session lasted one hour and included an opportunity for caregivers to discuss their feelings about oral health, coverage of the role of plaque in oral disease, and demonstrations of brushing techniques for dentures and natural teeth **Month 1:** Oral health care knowledge and attitudes assessed **Month 6**: Oral health care knowledge and attitudes assessed **Control:** Not received education	At month 6, the intervention group significantly improved dental health knowledge (*P* < 0.003) and attitude (*P* < 0.001), compared with control group **Conclusion**: The OHCE was well received and resulted in improved oral health care knowledge and attitudes
2	Frenkel HF (2001) [38]	Cardiff, UK	Cluster-RCT	All: *n* = 412 Mean age: 84.4 y Female: *n* = 323 Male: *n* = 89	I: *n* = 201 C: *n* = 211	**Intervention**: Caregivers in intervention group received oral health care education program (OHCE) **Baseline**: A Health Promoter presented the intervention Each session lasted 1 h and covered the role of plaque in oral disease, demonstrations of cleaning techniques for dentures and natural teeth **Month 1,6**: Main outcome measures were denture plaque, denture-induced stomatitis, dental plaque and gingivitis **Control**: Not received education	At month 6, the intervention group significantly improved oral health scores, and significantly reductions in denture plaque scores, denture-induced stomatitis prevalence compared to the control group (*P* < 0.0001) **Conclusion**: For a modest cost, OHCE can improve caregivers’ knowledge, attitudes and oral health care performance for older people, functionally dependent clients
3	Khanagar S (2014) [39]	India	RCT	All: *n* = 78 Age:18–40 y Female: *n* = 72 Male: *n* = 6	I: *n* = 38 C: *n* = 40	**Intervention**: Caregivers in intervention group received oral health care education) **Baseline**: The health educator gave a PowerPoint presentation on oral health to the caretakers and a live demonstration of oral hygiene techniques on study models. Also, a health education CD and manual were provided to the respective institutions **Month 6**: Main outcome measures were oral health knowledge of the caretakers **Control:** Not received education	At month 6, the intervention group significantly improved oral health knowledge compared with control group (*P* < 0.001) **Conclusion:** Educating the caretakers for assisting or enabling residents for maintaining oral hygiene is essential
4	Khanagar S (2015) [40]	India	Cluster RCT	All: *n* = 322 Age:18–40 y Gender isn't mentioned	I: *n* = 162 C: *n* = 160	**Intervention**: Caregivers in intervention group received oral health care education) **Baseline**: Oral hygiene status of older people residents was assessed by levels of debris, plaque of dentate and denture plaque, and denture stomatitis of denture wearing residents, respectively **Month 6**: Main outcome measures were levels of debris, plaque of dentate and denture plaque, and denture stomatitis of denture wearing residents, respectively **Control**: Not received education	At month 6, the intervention group significantly improved oral health knowledge compared with control group (*P* < 0.001) And significant reduction of plaque score, debris score, denture plaque score, denture stomatitis score (*P* < 0.001) **Conclusion:** There was a significant improvement in the oral-health knowledge among the caregivers and oral-hygiene status of the older people residents
5	Nicol R (2005) [44]	UK	Quasi experimental	All: *n* = 78 Age: ≥ 65 y Female: *n* = 63 Male: *n* = 15	Group I: *n* = 39 Group II: *n* = 39	**Intervention**: Caregivers in intervention group II received intensive training in mouth care based upon a resource pack entitled "Making Sense of the Mouth" containing a videotape, CD-ROM and full color pocket book **Baseline**: training sessions were undertaken for groups of six during working hours and lasted for approximately 90 min. An introductory 30-min lecture illustrating the mouth in health and disease (seven protocols on basic mouth care procedures) **Month 3,9**: Main outcome measures were Oral hygiene frequency**,** oral mucosal disease, angular cheilitis, Denture hygiene, Denture wearing habits, denture stomatitis **Month 18:** Final oral health assessment of all participants **Control**: After assessment of all patients at 9 months, training was provided to cares of patients in group I	At month 18, the intervention group (group II) significantly reduction in the number of residents left to undertake their own oral care (*P* < 0.001), significant improvements in denture hygiene and a reduction in the number of residents wearing dentures overnight (*P* < 0.001). The prevalence of oral mucosal disease dropped, with significant reductions in angular cheilitis and denture stomatitis (*P* < 0.001), compared with control group (group I) **Conclusion**: This education program was effective in changing oral health care procedures within long-stay institutions for the older people, with measurable improvements in oral health of the resident
6	Schwindling FS (2018) [42]	Germany	RCT	All: *n* = 269 Mean age:83.3 y Female: *n* = 189 Male: *n* = 80	I: *n* = 178 C: *n* = 91	**Intervention**: In the intervention group, caregivers were given oral health education, and ultrasonic cleaning devices were provided to clean removable prostheses **Baseline**: A PowerPoint lecture was given with the purpose of improving knowledge of oral health care and prevention of oral diseases The topics of the lecture: common oral problems in geriatric dentistry, brushing techniques for teeth and prostheses, handling of interdental space brushes and advice on other auxiliaries (for example mouth rinses) practical training with different types of prosthetic restoration was performed by use of typodonts **Month 6,12**: Main outcome measures were Plaque Control Record (PCR), Gingival Bleeding Index (GBI), Community Periodontal Index of Treatment Needs (CPITN) and Denture Hygiene Index (DHI) **Control**: Not received education	At month 12, the intervention group significantly improved PCR and DHI, compared with control group (*P* < 0.001) **Conclusion**: Education of caregivers improves and maintains the oral health of care dependent nursing home residents over longer periods. Use of ultrasonic devices is a promising means of improving denture hygiene among the severely care-dependent. Such interventions can be easily and cheaply implemented in routine daily care
7	Seleskog B (2018) [43]	Sweden	RCT	All: *n* = 66 Mean age: 88.5 y Female: *n* = 65 Male: *n* = 1	I: *n* = 33 C: *n* = 33	**Intervention**: Caregivers in intervention group received oral health care education) **Baseline**: Interventions included weekly theoretical and hands-on guidance from dental hygienists on oral hygiene procedures and discussions on oral care routines **Month 3**: Main outcome measures were residents’ oral health**,** dental plaque and gingival bleeding, Attitudes the staff to oral health care **Control**: Oral care was performed as usual	At month 3, the intervention group significantly improved Revised Oral Assessment Guide gums and lips scores showed a tendency to decrease, plaque levels improved significantly and a trend towards less gingival bleeding was observed compared with control group (*P* < 0.05) **Conclusions**: The oral healthcare situation for older people today is so complex that theoretical education at the group level regarding different aspects of oral health is not sufficient. Individual hands-on guidance by dental hygienists on a regular basis in everyday care may be a new approach
8	Paulsson G (1998) [45]	Sweden	Quasi-experimental	All: *n* = 2882 Age and gender are not mentioned	I: *n* = 1816 C: *n* = 1066	**Intervention**: Caregivers in intervention group (HHCE & LHCE) received oral health education program (OHEP) **Baseline**: The instruction material for the OHEP was thoroughly demonstrated and discussed: One series of slides (120 pictures), one videotape, and the compendium” oral health care knowledge for nursing personnel” were produced by one of the authors **12 months**: Main outcome measures were attitude to oral Hygiene, ability to handle, implementation Possibilities, knowledge of importance **Control:** (LHCE group) received oral health education program	After the OHEP, the HHCE group significantly improved their ability to perform oral hygiene for care receivers compared with the LHCE group (*P* < 0.01) **Conclusion:** oral health education program, offered to nursing personnel in special housing for the older people, positively affected the personnel’s ability to perform oral hygiene procedures for care receivers by improving attitude factors
9	MacEntee MI (2007) [41]	Canada	RCT	All: *n* = 152 Age: 79.1 y Gender is not mentioned	I: *n* = 59 C: *n* = 68	**Intervention**: Care-aides in the active group participated with a full-time nurse educator in a seminar about oral health care, and had unlimited access to the educator for oral health-related advice throughout the 3-month trial **Baseline**: The dental hygienist trained the nurse by discussing an annotated series of clinical photographs and a text summarizing the appearance and management of the more usual oral diseases encountered among frail elders **Month 3**: Main outcome measures were oral hygiene, gingival health, masticatory potential, Body Mass Index and Malnutrition Indicator Score, and asked to report on chewing difficulties **Control**: Care-aides in the control group participated in a similar seminar with a dental hygienist but they received no additional advice	At month 3, the intervention group were not significantly different from baseline in either group, indicating that education neither influenced the oral health nor the dental hygiene of the residents **Conclusions:** A pyramid-based educational scheme with nurses and care-aides did not improve the oral health of frail elders in this urban sample of LTC facilities

*Note: n* Number, *G* Group, *I* Intervention, *C* Control or Comparison, *RCT* Randomized Controlled Trial

### Design of the studies

In 14 studies, interventions were related to older people [23–32]. The ten studies were randomized controlled trials (RCT) studies [23–32] and four studies had quasi-experimental design [33–36]. Of the 23 included studies, nine studies were related to the caregivers of older people [37–43], that seven studies had RCT design [37–43] and two studies were quasi-experimental studies [44, 45]. Totally, there were 17 randomized controlled trials (RCT) studies [23–32, 37–43] and six studies had quasi experimental design [33–36, 44, 45].

### Study time and settings

Nine studies were published in 2018 or later [23–25, 30, 31, 33, 36, 42, 43]. Four studies were carried out in the UK [29, 37, 38, 44], three studies in Thailand [23, 28, 35], three in South Korea [24, 25, 33], two in USA [27, 31], two in Sweden [43, 45], two in India [39, 40], one in Japan [26], one in Australia [34], one in Taiwan [36], one in China [32], One in Iran [30], one in Germany [42], and one in Canada [41].

### Participants and follow-up duration

Most studies had a sample size less than 200 [23–26, 28, 31, 33–36, 39, 43, 44]. Fifteen studies were conducted among older people [23–36]. Nine studies focused on caregivers of older people [37–45]. The follow-up duration for one study was 36 months [27], one study 18 months [44], three studies were 12 months [31, 42, 45], one study nine months [44], six studies six months [28, 37–40, 42], six studies three months [23, 28, 35, 41, 43, 44], one study two months [29], three studies six weeks [24, 25, 33], four studies one month [30], and four studies without any follow-up [26, 32, 34, 36]. In fact, one study had three follow-ups [44] and five studies had two follow-ups [23, 28, 37, 38, 42].

### Theoretical framework usage

Theoretical frameworks have been used only in interventions related to older people. Of all the included studies, only 13% of them used theoretical framework. These studies include the Health Belief Model (HBM) in the study of Keyong et al., [23], adult learning theory in the study of Shokouhi et al., [30], and Social Cognitive Theory (SCL) in the study of Mariño et al. [34]. The Health Belief Model (HBM) as a conceptual framework in health education research was applied to improve self-management. The HBM can to predict behaviors according to constructs such as perceived susceptibility (person’s belief about chances of getting a disease or harmful situation), perceived severity (person’s belief about danger of a disease or harmful situation), perceived benefits (person’s belief regarding benefits to risk reduction of getting a disease or harmful situation), perceived barriers (person’s belief regarding costs of new behavior), cues to action (feel the necessity to take action), and self-efficacy (feel confident for the ability to perform a behavior) [46]. The adult learning theory refers to an organized process for raising the awareness, cognition, and skills of adults in order to be able to move towards excellence and evolution. The experience of people in the learning process and adults’ desire to learn without any compulsion are an important role in this theory [47]. The Social Cognitive Theory (SCL) helps to explain the interaction of the individual, environment, and behavior on behaviors [48]. The results of a review of eHealth intervention revealed that the majority of studies were based on SCT [47].

### Types of intervention

In the included studies of this review, educational interventions have been used for changing behavior or improving attitudes and increase awareness of oral health. In this review, educational interventions were provided for both older people [23–36] and caregivers [37–45]. In the related interventions to older people, different educational methods have been used such as lectures [23, 26–33, 36], mobile apps [24, 25], workbooks [25, 33], web based [34], educational video [28], motivational interviewing [30, 31, 35], and sending educational messages [30].

Also, the related interventions to caregivers included lectures [37–45], a live demonstration of oral hygiene techniques on study models [39], to provide oral health education CD and manual to the respective institutions [39], a videotape about oral health [44, 45], CD-ROM and full color pocket book about intensive training in mouth care [44], practical training with different types of prosthetic restoration by using typodonts [42], and hands-on guidance about oral hygiene procedures and discussions on oral care routines [43]. In one study, multifaceted programs including in-person training (individual training and group discussion) and non-attendance training (sending educational messages) were used [30].

### Types of outcome measures

From the results of 23 reviewed studies, 20 studies used self-reports as one of the outcome measurement methods [23–26, 28–40, 42, 43, 45]. In the related interventions to older people, the self-report measured variables included attitude [34, 35], knowledge [23–25, 27, 31, 33–35], oral health perceptions [23, 25], oral health recognition [33], self-efficacy [31, 34], oral health related quality of life (OHRQoL) [30, 31], oral health literacy [36], practices [34, 35], and skills of oral health [23–25, 27, 33–35]. In the related interventions to caregivers, the self-report measured variables included attitude [37, 38, 43, 45], knowledge [37–40, 42, 45], and performance of oral health [38].

Also, in the reviewed studies, objective measures were used to evaluate the effects of interventions. Objective measures are contained below:

1) In interventions related to older people, these items included tongue pressure, unstimulated salivary flow rate [24], resting salivation in the second and third cumulated Repetitive Saliva Swallowing Test times [26], plaque score [23, 28, 35], clinical attachment level (CAL) [35], gingival inflammation [23], clinical attachment loss [23, 28], percentage of bleeding on probing (BOP) [35], probing depth [28], root surfaces with new caries [28], active root caries surfaces [28], subjective oral dryness [24], O’Leary index [25, 33], tongue coating index [25, 33], bitterness threshold [26], coronal caries events [27], root caries events [27], gingival index score [28, 35], pocket depth [35], and glycemic indexes (glycosylated hemoglobin (HbA1c) and fasting plasma glucose (FPG)) [28, 35].

2) In interventions related to the caregivers, the items of objective measures included oral health scores [38, 45], denture hygiene [44], plaque control record [42], Denture Hygiene Index (DHI) [42], plaque levels [43], denture plaque score [38, 40], denture stomatitis score [38, 40, 44], debris score [40], the number of residents wearing dentures overnight [44], oral mucosal disease [44], angular cheilitis [44], revised oral assessment guide gums and lips scores [43], gingival bleeding [43], and plaque score [40].

### The effects of interventions

In the related interventions to older people, the self-report measured variables improved including attitude [34, 35], knowledge [23–25, 27, 31, 33–35], oral health perceptions [23, 25], oral health recognition [33], self-efficacy [31, 34], oral health related quality of life (OHRQoL) [30, 31], oral health literacy [36], practices [34, 35], and skills of oral health [23–25, 27, 33–35]. In the related interventions to caregivers, the self-report measured variables improved including attitude [37, 38, 43, 45], knowledge [37–40, 42, 45], and performance of oral health [38].

The interventions in older people and caregivers have led to improvement or decreasing the below objective measures.

In the related interventions to older people, the improved objective measures included tongue pressure, unstimulated salivary flow rate [24], resting salivation in the second and third cumulated Repetitive Saliva Swallowing Test times [26] and decreased items included plaque score [23, 28, 35], clinical attachment level (CAL) [35], gingival inflammation [23], clinical attachment loss [23, 28], percentage of bleeding on probing (BOP) [35], probing depth [28], root surfaces with new caries [28], a great number of active root caries surfaces [28], subjective oral dryness [24], O’Leary index [25, 33], tongue coating index [25, 33], bitterness threshold [26], coronal caries events [27], root caries events [27], gingival index score [28, 35], pocket depth [35], and glycemic indexes (glycosylated hemoglobin (HbA1c), fasting plasma glucose (FPG)) [28, 35].

In the related interventions to caregivers, the improved objective measures included the following: oral health scores [38, 45], denture hygiene [44], plaque control record (PCR) [42], denture hygiene index (DHI) [42], and plaque levels [43], and the objective measures included reduction of items such as denture plaque score [38, 40], denture stomatitis score [38, 40, 44], debris score [40], the number of residents wearing dentures overnight [44], oral mucosal disease [44], angular cheilitis [44], revised oral assessment guide gums and lips scores.

[43], gingival bleeding [43], and plaque score [40]. In one study, intervention group was not significantly different from baseline [41].

### Risk of bias of the included studies

We did not exclude studies based on the results of the quality assessment. Inter-rater agreement varied across EPHPP components ratings. Overall, there was a good agreement between the two reviewers (Kappa coefficient = 0.80, *p* < 0.001). Fifteen studies of the included studies were of moderate quality and eight studies were weak quality (Table 3).

**Table 3:** Quality assessment using EPHPP quality rating

Author, year	Selection bias	Study design	Blinding	Confounders	Data collection methods	Withdrawal /dropouts	Study quality
Ki, 2021 [24]	Moderate	Strong	Weak	Moderate	Moderate	Strong	Weak
Lee, 2021 [25]	Moderate	Strong	Weak	Weak	Strong	Strong	Weak
Sun, 2021 [36]	Moderate	Moderate	Strong	Weak	Strong	Strong	Moderate
Lee, 2020 [33]	Moderate	Moderate	Weak	Weak	Strong	Strong	Moderate
Shokouhi, 2020 [30]	Weak	Strong	Strong	Moderate	Strong	Strong	Moderate
Keyong, 2019 [23]	Strong	Strong	Strong	Weak	Moderate	Strong	Moderate
Tellez, 2019 [31]	Strong	Strong	Weak	Strong	Strong	Strong	Moderate
Schwindling, 2018 [42]	Strong	Strong	Weak	Strong	Strong	Moderate	Moderate
Seleskog, 2018 [43]	Weak	Strong	Strong	Strong	Moderate	Strong	Moderate
Marino, 2016 [34]	Moderate	Moderate	Weak	Weak	Moderate	Weak	Weak
Khanagar, 2015 [40]	Strong	Strong	Weak	Moderate	Strong	Strong	Moderate
Ohara, 2015 [26]	Strong	Strong	Weak	Weak	Strong	Strong	Weak
Saengtipbovorn, 2015 [28]	Moderate	Strong	Weak	Weak	Strong	Strong	Weak
Khanagar, 2014 [39]	Strong	Strong	Weak	Moderate	Strong	Strong	Moderate
Saengtipbovorn, 2014 [35]	Moderate	Moderate	Moderate	Strong	Strong	Weak	Moderate
MacEntee, 2007 [41]	Strong	Moderate	Strong	Weak	Strong	Strong	Moderate
Zhang, 2007 [32]	Moderate	Strong	Strong	Weak	Moderate	Strong	Moderate
Nicol, 2005 [44]	Moderate	Moderate	Strong	Weak	Strong	Strong	Moderate
Frenkel, 2002 [37]	Strong	Strong	Strong	Moderate	Weak	Weak	Weak
Frenkel, 2001 [38]	Strong	Strong	Strong	Moderate	Weak	Strong	Moderate
Powell, 1999 [27]	Moderate	Strong	Moderate	Weak	Strong	Strong	Moderate
Paulsson, 1998 [45]	Strong	Moderate	Weak	Weak	Strong	Moderate	Weak
Schou, 1989 [29]	Strong	Strong	Strong	Weak	Weak	Weak	Weak

## Discussion

To the best of our knowledge, there are no systematic reviews to identify the types of health education interventions and to determine their effects on oral and dental health among older people. As mentioned previously, older people have many oral and dental problems that can negatively affect their physical or psychosocial health [14, 15]. This situation is exacerbated in some older people, such as older people with cognitive impairment/ dementia. Evidence shows that cognitive impairment and dementia influence oral-dental health and these disorders lead to the reduction of dental service use. According to the study of Jockusch et al., with increasing cognitive impairment/dementia among older people, there was a significant difference in the number of decayed teeth. Also, with increasing dementia, the degree.

of restoration decreased and oral/denture hygiene declined significantly [49]. So, studies that have done interventions for the oral and dental health of older people with cognitive impairment/ dementia disorders were excluded from this review. The results of the current study demonstrated that the majority of the included studies had randomized controlled trials design (17/23). Eight studies (34%) were categorized as low quality. As is clear, randomized clinical trials are the best method for controlling selection and confounding biases [50, 51]. Quasi-experimental designs, due to the lack of random allocation, cannot express the effect of an intervention as clearly as experimental studies [52]. In this review, although 17 studies were randomized controlled trials, six of them were of low quality. It seems that in the future studies in the field of oral and dental health of older people, it is necessary to conduct more high-quality randomized clinical trial studies. In this review, it was found that theoretical frameworks had been used in only three interventions related to older people (13%). These studies include the Health Belief Model (HBM) in the study of Keyong et al., [23], adult learning theory in the study of Shokouhi et al., [30], and Social Cognitive Theory (SCL) in the study of Mariño et al. [34]. Evidence indicates that interventions aimed at changing or modifying behavior would be more effective if they are designed and implemented based on a suitable theoretical frameworks [53, 54], because theoretical frameworks offer a systematic approach to a better understanding of phenomena by providing explanations related to why and under what conditions. In other words, for more effectiveness of educational programs, it is recommended to use theoretical frameworks of health education and health promotion [55]. Results demonstrated that using the mentioned theoretical frameworks led to improved oral health perception, behavior, and oral health status [23], improved oral health knowledge, attitudes, and self-efficacy [34], and improved oral health-related quality of life among older people [30]. In this review, we could not discuss in detail the quality and effectiveness of framework-based interventions for two reasons: a) Frameworks were used in only three studies. b) Due to the use of different frameworks in the design of interventions, the outcomes were not the same, so that we could compare them.

Also, the results of this review show that although a variety of interventions were used in the reviewed studies, more lectures were used in the interventions related to older people [23, 26–33, 36]. In the interventions related to caregivers, in addition to lectures [37–45], practical training was also used [39, 42–44]. Some of the educational lectures in the interventions related to older people or caregivers were: oral hygiene instruction, facial and tongue muscle exercise, and salivary gland massage [26], and toothbrushing with fluoride toothpaste, cleaning dentures, and self-check oral health [28], and the importance of oral health, common oral health problems among older people (coronal and root dental caries, gingivitis, periodontitis, oral cancer), and oral hygiene self-care (flossing, brushing, rinsing, and denture care) [31]. Also, some of the practical education included a live demonstration of oral hygiene techniques on study models [39], brushing techniques for teeth/prostheses, and handling of interdental space brushes [42], and tooth brushing, denture care, and a variety of oral hygiene aids [44]. In fact, the majority of interventions used traditional methods for education, and only one study used web-based oral health presentations for the older people [34]. The finding of a review of interventional studies in Iran about investigating the effect of different educational methods in preventing disease in elderly people showed that no study had used electronical interventions using social networking software (Telegram, WhatsApp, etc.), web-based, or e-mail-based interventions. In other words, all studies had used traditional approaches for modifiying lifestyle and promoting health behaviors [56]. It seems that although the traditional methods of education are more pleasant and comfortable for older people and even caregivers, in the digital age, it is necessary to use new technologies in the education of older people. Indeed, the reduced use of new technologies by older people compared to other age groups has caused the digital divide. One of the effective ways to overcome this problem is to help older people accept new information and communication technologies [57]. The evidence shows that various theoretical frameworks have been used to accept technology in older people, such as diffusion of innovations [58], theory of reasoned action [59], and theory of planned behavior [56, 60]. The use of new technologies not only provides support services such as remote care for older people, but also improves their quality of life and individual independence. Further studies are recommended in the area of educational methods and comparison of these methods [56].

From the results of 23 reviewed studies, 20 studies used self-reports as one of the outcome measurement methods [23–26, 28–40, 42, 43, 45]. In interventions related to older people, more self-report variables were measured than interventions related to caregivers. In the related interventions to caregivers, the self-report measured variables included attitude [37, 38, 43, 45], knowledge [37–40, 42, 45], and performance of oral health [38], which all self-report measured variables were improved compared to before the intervention. In the interventions related to older people and caregivers, a wide variety of objective outcomes were measured, so it was practically impossible to compare the outcomes of the interventions. Only plaque score was measured both in interventions related to older people [23, 28, 35] and in interventions related to caregivers [40]. In the study of Khanagar et al., (2015) led to a significant reduction of mean plaque score from a baseline score of 3.17 ± 0.40 to 1.57 ± 0.35 post-intervention (six-month) [40]. Also, in the studies of keyong et al., (2019), Saengtipbovorn et al., (2015), and Saengtipbovorn et al., (2014) in older people led to a significant reduction of mean plaque score at baseline score compared to post-intervention (3.28 ± 1.03 Vs. 2.69 ± 0.56, 0.04 ± 0.07 Vs. 0.23 ± 0.07, and 0.59 ± 0.42 Vs. 0.26 ± 0.31, respectively). These results show that the reduction of the mean plaque score in the intervention related to caregivers was reported more than the interventions related to older people. It seems that considering this index in interventions related to caregivers will be more effective. Dental plaque is a biofilm of microorganisms on the tooth surface that plays an important role in the spread of caries and periodontal disease [61]. Gram-positive and gram-negative bacteria that are present on the surface of dental plaque can cause gingivitis and, if left untreated, can create periodontitis [62]. Some factors such as poor and insufficient oral health status and the use of prosthesis lead to promote the creation and accumulation of plaque in older people [63]. Plaque control is an effective way to treat and prevent gingivitis and is an essential part of all methods of treating and preventing periodontal diseases [64]. Although mechanical control of plaque is the most reliable method of oral hygiene, plaque control by brushing alone is not enough to control periodontal diseases [65]. The use of chemical substances such as mouthwashes, gel and antimicrobial toothpaste is of particular importance [66, 67]. The results of a current scoping review demonstrated that mechanical, chemical and educational strategies are effective in dental plaque control in older people [68].

### Strengths and limitations

The most important strength of this study was that the current study was the first systematic review in order to identify the types of health education interventions and to determine their effects on oral and dental health in older people. Considering the role of interventions in improving the oral and dental health of older people in the reviewed studies, it seems that interested researchers can use the experiences of these studies in the design and implementation of interventions according to the characteristics of their studied society. This review had some limitations. First of all, we included only studies in English. The second limitation was the lack of access to the full text of some articles. The third limitation was that although the current study includes numerous RCTs, many of them have very low sample size and imbalance in the sample size of the studied groups. It is possible that, despite being RCTs, the strength of evidence is less than ideal. Finally, the results may have a degree of selection bias because of ignoring gray literature, unpublished studies, and studies published in other databases.

## Conclusion

This review provides evidence that the use of oral and dental health educational interventions was effective in improving the oral health of older people. Educational interventions were carried out both among older people and among their caregivers. Although a variety of interventions were used in the reviewed studies, more lectures were used in the interventions related to older people. In the interventions related to caregivers, in addition to lectures, practical education was also used. It is recommended to perform higher quality studies for assessing the effectiveness of interventions in this field.

### Supplementary Information

**Additional file 1. **The search strategy for PubMed/Medline.

## Data Availability

All supporting data is available through the corresponding author.
