# Correction: The impact of health education interventions on oral health promotion among older people: a systematic review

**DOI:** 10.1186/s12877-023-04351-w

**Published:** 2023-12-05

**Authors:** Saeid Bashirian, Sahar Khoshravesh, Erfan Ayubi, Akram Karimi-Shahanjarini, Samane Shirahmadi, Parshang Faghih Solaymani

**Affiliations:** 1 Autism Spectrum Disorders Research Center, Hamadan University of Medical Sciences, Hamadan, Iran; 2 Department of Community Health Nursing, School of Nursing and Midwifery Hamadan University of Medical Sciences, Hamadan, Iran; 3 Chronic Diseases (Home Care) Research Center, Hamadan University of Medical Sciences, Hamadan, Iran; 4 Cancer Research Center, Hamadan University of Medical Sciences, Hamadan, Iran; 5 Social Determinants of Health Research Center, Hamadan University of Medical Sciences, Hamadan, Iran; 6 Department of Community Oral Health, School of Dentistry, Dental Research Center, Hamadan University of Medical Sciences, Hamadan, Iran

**BMC Geriatrics (2023) 23:548**

10.1186/s12877-023-04259-5

After publication of this article [1], the authors reported that the affiliation details for the 3rd, 4th and 6th author were incorrect. The correct author-affiliation information is as follows:

 Saeid Bashirian^1^, Sahar Khoshravesh^2,3^, Erfan Ayubi^4,5^, Akram Karimi Shahanjarini^5^, Samane Shirahmadi^6^ and Parshang Faghih Solaymani^5^*

^1^Autism Spectrum Disorders Research Center, Hamadan University of Medical Sciences, Hamadan, Iran

^2^Department of Community Health Nursing, School of Nursing and Midwifery, Hamadan University of Medical Sciences, Hamadan, Iran

^3^Chronic Diseases (Home Care) Research Center, Hamadan University of Medical Sciences, Hamadan, Iran

^4^Cancer Research Center, Hamadan University of Medical Sciences, Hamadan, Iran

^5^Social Determinants of Health Research Center, Hamadan University of Medical Sciences, Hamadan, Iran

^6^Department of Community Oral Health, School of Dentistry, Dental Research Center, Hamadan University of Medical Sciences, Hamadan, Iran

The original article [1] has been corrected.
