# Supplementary material for: The impact of health education interventions on oral health promotion among older people: a systematic review
Source: BMC Geriatr. 2023 Sep 11;23:548. doi: 10.1186/s12877-023-04259-5 (PMC10494401; doi:10.1186/s12877-023-04259-5)
Supplement: Supplementary file 1 — Additional file 1. The search strategy for PubMed/Medline. [file 12877_2023_4259_MOESM1_ESM.docx]

**The search strategy for PubMed/Medline**

("dental"[Title/Abstract] OR "oral"[Title/Abstract]) AND ("health education"[Title/Abstract] OR "health promotion"[Title/Abstract]) AND ("elderly"[Title/Abstract] OR "aging" [Title/Abstract] OR "older adults"[Title/Abstract] OR "aged"[Title/Abstract] OR "older"[Title/Abstract] OR "elder"[Title/Abstract]) AND ("randomized controlled trials"[Title/Abstract] OR "clinical trials"[Title/Abstract] OR "trial"[Title/Abstract] OR "quasi experimental"[Title/Abstract] OR "non randomized controlled trials"[Title/Abstract])
